# A large esophageal gastrointestinal stromal tumor that was successfully resected after neoadjuvant imatinib treatment: case report

**DOI:** 10.1186/1477-7819-12-47

**Published:** 2014-02-27

**Authors:** Senichiro Yanagawa, Kazuaki Tanabe, Takahisa Suzuki, Noriaki Tokumoto, Koji Arihiro, Hideki Ohdan

**Affiliations:** 1 Department of Gastroenterological and Transplant Surgery, Applied Life Sciences, Institute of Biomedical and Health Sciences, Hiroshima University, 1-2-3 Kasumi, Minami-ku, Hirohima 734-8551, Japan; 2 Department of Anatomical Pathology, Hiroshima University Hospital, 1-2-3 Kasumi, Minami-ku, Hirohima 734-8551, Japan

**Keywords:** Esophageal GIST, Neoadjuvant chemotherapy, Imatinib

## Abstract

A 49-year-old man was admitted to our hospital with a 1-month history of dysphagia. An upper endoscopy revealed a lower esophageal submucosal tumor. Immunohistochemical staining of the biopsy specimen revealed KIT positivity. Thus, the tumor was diagnosed as a gastrointestinal stromal tumor (GIST). After 6 months of imatinib treatment, the tumor decreased from 92 mm × 55 mm × 80 mm to 65 mm × 35 mm × 55 mm in diameter, and surgery was performed. The tumor was completely resected without rupture, by partial esophagogastric resection through a thoracotomy incision, using an abdominal laparoscopic approach. Immunohistochemical staining revealed that the tumor was negative for c-kit but positive for CD34. Genetic examination showed that the tumor had a mutation in exon 11. The patient experienced minor leakage but recovered conservatively. Adjuvant imatinib was initiated 64 days after surgery. We report this rare case to show the potential of preoperative imatinib treatment in patients with large esophageal GISTs, to achieve complete resection without rupture.

## Background

Gastrointestinal stromal tumors (GISTs) are mesenchymal tumors of the gastrointestinal tract, commonly found in the stomach (60% to 70%) and small intestine (20% to 30%) [1,2]. Surgery is the first treatment for resectable GISTs, which cures approximately 60% of patients [3]. However, the prognosis of patients who relapse is poor. Imatinib mesylate is now the standard drug for patients with unresectable or metastatic GISTs and was recently approved for use as an adjuvant treatment [4,5]. Currently, there is no evidence of an effective neoadjuvant therapy for GISTs, especially for very large primary GISTs, which have an increased risk of a positive resection margin [6]. As esophageal GISTs are very rare, accounting for <2% of all GISTs [2,7], the available literature is limited. Moreover, the use of imatinib therapy followed by surgery has been published only for a few cases [8]. We report a large esophageal GIST in a 49-year-old man who underwent surgery after neoadjuvant chemotherapy.

## Case presentation

**Figure 1:**
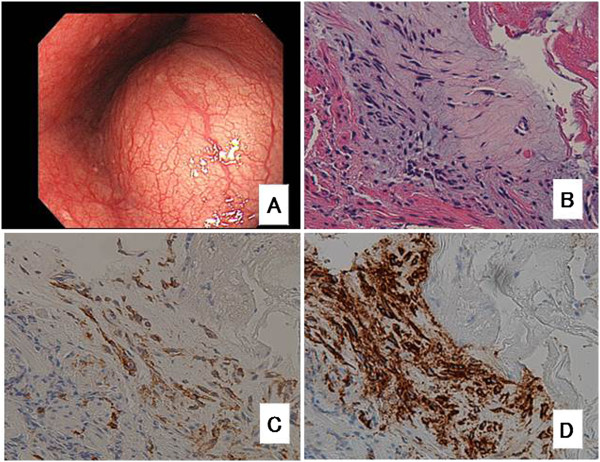
**Preoperative image and tumor biopsy images. (A)** Endoscopic image taken before chemotherapy showing the tumor with a smooth surface in the lower esophagus. Histopathologically, H & E staining showed that the spindle tumor cells had no mitotic activity, as shown at × 200 **(B)**. The tumor cells were positive for c-kit, as shown at × 200, **(C)** and CD34, as shown at × 200, **(D)** using immunohistochemistry.

**Figure 2:**
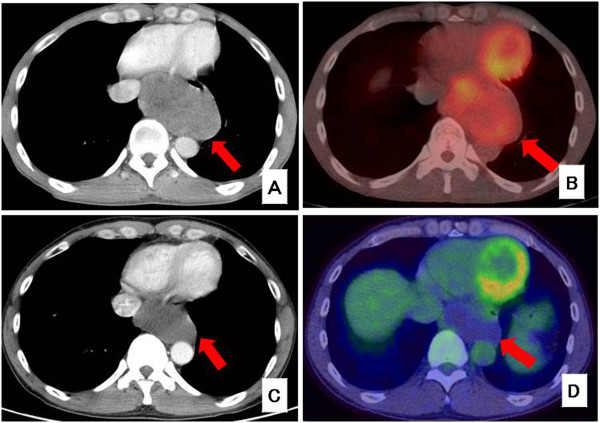
**CT and PET-CT images. (A)** CT scan before chemotherapy. The tumor was 92 × 55 mm × 80 mm in diameter. **(B)** PET-CT scan before chemotherapy. SUV max was 4.9. **(C)** CT scan after chemotherapy. The tumor was 70 mm × 37 mm × 60 mm in diameter. **(D)** PET-CT scan after chemotherapy. SUV max was 1.2.

A 49-year-old man was admitted to our hospital with a 1-month history of dysphagia. He did not have any history of illness or any significant family history. Physical examination revealed no abnormalities. Standard laboratory test results on serum and urine showed no significant findings. Tumor markers such as carcinoembryonic antigen, carbohydrate antigenic determinant 19-9 and squamous cell carcinoma antigen were also within the reference limits. An upper endoscopy revealed a submucosal tumor with a narrowed lumen in the lower esophagus (Figure 1A). After a fine-needle aspiration biopsy, the tumor was diagnosed as a GIST, characterized pathologically by H & E staining and immunostaining (Figure 1B,C,D). A computed tomography (CT) scan showed a mass, 92 mm × 55 mm × 80 mm in diameter (Figure 2A), at the lower esophagus and the maximum standardized uptake value (SUV max) was 4.9 on a positron emission tomography (PET)-CT scan (Figure 2B). There was no apparent distant metastasis. The tumor was classified as intermediate risk according to Fletcher’s classification [9]. It was judged to be resectable, but with a risk for rupture during resection because of the size and localization of the lesion. Therefore the patient received neoadjuvant imatinib (400 mg/day) treatment and was followed-up for 2 months with CT scans to assess the therapeutic effect. He was treated for 6 months with chemotherapy and the tumor shrank by 25% (Figure 2C). A PET-CT scan showed a reduction in SUV max to 1.2 (Figure 2D).

**Figure 3:**
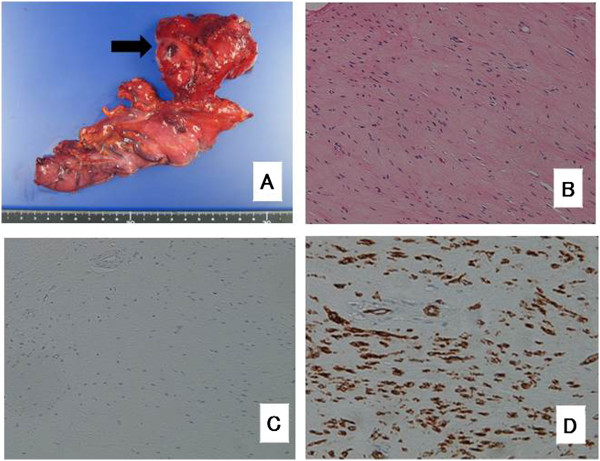
**Resected surgical specimen. (A)** The resected tumor was approximately 65 mm × 35 × 55 mm in diameter. **(B)** Histopathologically, H & E staining showed that the tumor cells had no necrosis and mitotic activity, as shown at × 200. The tumor cells were negative for c-kit, as shown at × 200, **(C)** and positive for CD34, as shown at × 200, **(D)** using immunohistochemistry.

The patient underwent surgery after the chemotherapy. The surgical procedure was a partial esophagogastric resection through a thoracotomy incision, using an abdominal laparoscopic approach. A lymphadenectomy was not performed. On gross examination, the resected tumor was soft and had shrunk to 65 mm × 35 mm × 45 mm in diameter (Figure 3A). The esophageal mucosa appeared normal without ulcerations. Histological analysis showed that the tumor consisted of spindle cells with no mitotic activity (Figure 3B). Immunohistochemical staining revealed that the spindle cells were negative for c-kit (Figure 3C) but positive for CD34 (Figure 3D). Genetic examination showed that the tumor had an exon 11 mutation.

The patient experienced aspiration pneumonia and minor leakage after the operation, but recovered conservatively from both conditions. He was discharged 52 days after the surgery, and adjuvant chemotherapy (imatinib, 400 mg/day) was initiated at 64 days after the surgery. There has been no recurrence for 12 months post-surgery.

## Discussion

Complete surgical resection is the standard treatment for localized GISTs; however, 40% to 90% of curative resection patients experience recurrence [10]. Esophageal GISTs are rare, highly vascularized tumors. Large tumors specifically have a tendency to rupture or have a risk of a positive margin despite a macroscopically complete resection. Tumor rupture or the presence of a residual tumor is strongly associated with recurrence and poor prognosis [11,12].

The biological potential of stomach or small intestinal GISTs is related to their size and mitotic activity, which may also be true of esophageal GISTs. The prognosis is commonly stratified according to the Joensuu risk criteria [3] and accurate risk stratification is important in considering adjuvant imatinib therapy. It has been established that 3 years of adjuvant imatinib is beneficial for recurrence-free survival and overall survival for-high risk GISTs [4].

Although the benefit of imatinib for malignant or high-risk GISTs is beyond doubt, the clinical outcome of neoadjuvant treatment has not yet been established. In our case, we were able to a perform complete resection of the GIST owing to the decreased tumor size after the treatment with imatinib. The tumor was diagnosed to be of intermediate risk according to Fletcher’s classification [9]. We used neoadjuvant imatinib (400 mg/day for 6 months) to achieve complete resection without rupture. In our case, there was no rupture during the operation and a pathologically negative margin was obtained. The appropriate duration of neoadjuvant therapy with imatinib is controversial. Most of the positive responses to chemotherapy occur within 6 months of administration and secondary mutations may occur after 10 months of treatment, hence some reports suggest 6 months of neoadjuvant chemotherapy for GISTs [13,14]. In our case, sufficient shrinkage of the tumor was attained after 6 months of treatment; therefore, a minimal resection could be performed during esophagectomy. The reason for the negative c-kit and positive CD34 findings, despite the tumor being positive for both markers preoperatively, is unclear. These biomarker changes may be because of the therapeutic effect of imatinib; however, whether these findings are indicative of a pathological complete response is uncertain. Tumors could show a completely altered morphology and immunophenotype after imatinib treatment; thus, pathologists should be aware of this phenomenon [15]. There have been several case reports demonstrating that a complete response was obtained with imatinib treatment even in patients with metastatic GISTs [16].

In summary, we reported a case of a large esophageal GIST that was successfully resected after imatinib treatment. Although the effects of neoadjuvant imatinib therapy on overall survival are not yet clear, long-term imatinib administration has the potential to promote curative surgery for some patients with large GISTs.

## Conclusions

We treated a case of a large esophageal GIST that was successfully resected after neoadjuvant imatinib treatment. When an esophageal GIST is a certain size, located in the narrow mediastinal space and is difficult to dissect, neoadjuvant imatinib treatment might be one of the options for a minimally invasive surgical resection without rupture.

## Consent

Written informed consent was obtained from the patient for publication of this case report and accompanying images. A copy of the written consent is available for review by the Editor-in-Chief of this journal.

## Abbreviations

CT: computed tomography; GIST: gastrointestinal stromal tumor; H & E: hematoxylin and eosin; OS: overall survival; PET: positron emission tomography; SUV max: maximum standardized uptake value.

## Competing interests

The authors declare that they have no competing interests.

## Authors’ contributions

SY and KT wrote the manuscript. SY, KT, TS and NT performed surgery. KA carried out the pathological examination. HO was involved in the final editing. All authors approved the final manuscript.

## References

[B1] Miettinen M Sarlomo-Rikala M Lasota J Gastrointestinal stromal tumours Ann Chir Gynaecol 1998 87 278 281 9891765

[B2] Miettinen M Lasota J Gastrointestinal stromal tumors: review on morphology, molecular pathology, prognosis, and differential diagnosis Arch Pathol Lab Med 2006 130 1466 1478 17090188 10.5858/2006-130-1466-GSTROM

[B3] Joensuu H Vehtari A Riihimaki J Nishida T Steigen SE Brabec P Plank L Nilsson B Cirilli C Bordoni A Magnusson MK Linke Z Sufliarsky J Federico M Jonasson JG Dei Tos AP Rutkowski P Risk of recurrence of gastrointestinal stromal tumour after surgery: an analysis of pooled population-based cohorts Lancet Oncol 2012 13 265 274 10.1016/S1470-2045(11)70299-6 22153892

[B4] Eisenberg BL The SSG XVIII/AIO trial: results change the current adjuvant treatment recommendations for gastrointestinal stromal tumors Am J Clin Oncol 2013 36 89 90 10.1097/COC.0b013e31827a7f55 23334483

[B5] Dematteo RP Ballman KV Antonescu CR Maki RG Pisters PW Demetri GD Blackstein ME Blanke CD Von Mehren M Brennan MF Patel S McCarter MD Polikoff JA Tan BR Owzar K American College of Surgeons Oncology Group (ACOSOG) intergroup Adjuvant GIST Study Team Adjuvant imatinib mesylate after resection of localised, primary gastrointestinal stromal tumour: a randomised, double-blind, placebo-controlled trial Lancet 2009 373 1097 1104 10.1016/S0140-6736(09)60500-6 19303137 PMC2915459

[B6] Raut CP Posner M Desai J Morgan JA George S Zahrieh D Fletcher CD Demetri GD Bertagnolli MM Surgical management of advanced gastrointestinal stromal tumors after treatment with targeted systemic therapy using kinase inhibitors J Clin Oncol 2006 24 2325 2331 10.1200/JCO.2005.05.3439 16710031

[B7] Lee HJ Park SI Kim DK Kim YH Surgical resection of esophageal gastrointestinal stromal tumors Ann Thorac Surg 2009 87 1569 1571 10.1016/j.athoracsur.2009.01.051 19379907

[B8] Blum MG Bilimoria KY Wayne JD de Hoyos AL Talamonti MS Adley B Surgical considerations for the management and resection of esophageal gastrointestinal stromal tumors Ann Thorac Surg 2007 84 1717 1723 10.1016/j.athoracsur.2007.05.071 17954092

[B9] Fletcher CD Berman JJ Corless C Gorstein F Lasota J Longley BJ Miettinen M O’Leary TJ Remotti H Rubin BP Shmookier B Sobin LH Weiss SW Diagnosis of gastrointestinal stromal tumors: a consensus approach Hum Pathol 2002 33 459 465 10.1053/hupa.2002.123545 12094370

[B10] Rossi CR Mocellin S Mencarelli R Foletto M Pilati P Nitti D Lise M Gastrointestinal stromal tumors: from a surgical to a molecular approach Int J Cancer 2003 107 171 176 10.1002/ijc.11374 12949790

[B11] Ng EH Pollock RE Munsell MF Atkinson EN Romsdahl MM Prognostic factors influencing survival in gastrointestinal leiomyosarcomas: implications for surgical management and staging Ann Surg 1992 215 68 77 10.1097/00000658-199201000-00010 1731651 PMC1242372

[B12] Rutkowski P Nowecki ZI Michej W Debiec-Rychter M Wozniak A Limon J Siedlecki J Grzesiakowska U Kakol M Osuch C Polkowski M Gluszek S Zurawski Z Ruka W Risk criteria and prognostic factors for predicting recurrences after resection of primary gastrointestinal stromal tumor Ann Surg Oncol 2007 14 2018 2027 10.1245/s10434-007-9377-9 17473953

[B13] Xia L Zhang MM Ji L Li X Wu XT Resection combined with imatinib therapy for liver metastases of gastrointestinal stromal tumors Surg Today 2010 40 936 942 10.1007/s00595-009-4171-x 20872196

[B14] Gold JS Dematteo RP Neoadjuvant therapy for gastrointestinal stromal tumor (GIST): racing against resistance Ann Surg Oncol 2007 14 1247 1248 10.1245/s10434-006-9291-6 17265116

[B15] Pauwels P Debiec-Rychter M Stul M De Wever I Van Oosterom AT Sciot R Changing phenotype of gastrointestinal stromal tumours under imatinib mesylate treatment: a potential diagnostic pitfall Histopathology 2005 47 41 47 10.1111/j.1365-2559.2005.02179.x 15982322

[B16] Akiyoshi T Oya M Fujimoto Y Kuroyanagi H Ueno M Yamaguchi T Takahashi S Hatake K Katori M Yamamoto N Muto T Complete resection after imatinib treatment of a gastrointestinal stromal tumor of the ileum with peritoneal metastases: report of a case Surg Today 2010 40 272 276 10.1007/s00595-008-4037-7 20180084

